# Endometriosis: A Review of Clinical Diagnosis, Treatment, and Pathogenesis

**DOI:** 10.7759/cureus.28864

**Published:** 2022-09-06

**Authors:** Saurabh Chauhan, Akash More, Vaishnavi Chauhan, Aditya Kathane

**Affiliations:** 1 Clinical Embryology, School of Allied Health Science, Datta Meghe Institute of Medical Sciences (DU), Wardha, IND; 2 Anatomy, Wardha Test Tube Baby Center, Datta Meghe Institute of Medical Sciences (DU), Wardha, IND

**Keywords:** infertility, endometrial, uterus, scar, endometriosis

## Abstract

Endometriosis is a condition that affects women of reproductive age, and it is distinguished by the development of endometrial-like tissue outside the uterine cavity. It is frequently accompanied by persistent pelvic discomfort and infertility. This investigation looks into recent findings on clinical manifestation to help doctors and improve women's health. PubMed and Google Scholar were used to review on clinical diagnosis of endometriosis. The search strategy contained the terms “endometriosis” and “clinical diagnosis.” All research articles published between 1960 and 2021 were included in the search. The findings were then categorized to summarize the evidence. There was a total of 29 instances of endometriosis discovered. The patients' ages varied from 20 to 45 years old, with a median of 28.8 years and a mean of 29.4±7.7 years. Dysmenorrhea is a common disorder among adolescent girls experiencing various physical and emotional symptoms which have a detrimental influence on their quality of life. In this study, scar endometriosis was shown to be the more common variety of endometriosis, with 50% of cases predominantly developing at the lower segment cesarean section (LSCS) scar site. As a result, women with endometriosis are more likely to have miscarriages, and the quality of their embryos declines as a result.

## Introduction and background

Endometriosis is the most perplexing gynecological condition [1-3]. Endometriosis influences 10%-15% of all reproductive-age females and 70% of women with persistent pelvic pain [4]. The ovaries and pelvic peritoneum are the most common sites for developing endometriotic lesions. Endometriotic lesions can also develop in other places including the fallopian tube, abdominal wall, bowels, cervix, bladder, and vagina [5,6]. The pathophysiology of endometriosis and pain is poorly known, with most gynecologists believing that inflammation is a crucial source of irritation in endometriosis [7]. After successful surgery, some women seem to be “cured” of the disorder, but the majority will have a recurrence of symptoms. Endometriosis costs roughly 1 to 2 lakhs per woman in India, according to a survey [8]. The uterosacral ligaments, the upper third part of the posterior vaginal wall, the rectovaginal space, the intestine, and the urinary system are frequently involved in the latter [9]. Researchers have previously looked at apoptosis, cell cycle changes, and granulosa cell oxidative stress as indicators of oocyte quality as a source of endometriosis subfertility [10-12].

Endometriosis is a female reproductive system disorder where the endometrium-like tissue develops outside of the uterus; it usually affects the ovaries and peritoneum, causing premenstrual discomfort and dysmenorrhea [13,14]. The most widely recognized explanation of endometriosis is that endometrial tissue is implanted in the peritoneal cavity by retrograde menstruation. The first theory describing the origin of endometriosis is the retrograde menstruation theory. According to this idea, endometriosis develops when sloughed endometrial cells and debris after menstruation travel retrogradely down the fallopian tubes and enter the pelvic cavity. 76%-90% of women having patent fallopian tubes experience retrograde menstruation, albeit not all of these women have endometriosis [15]. Endometrial cell resorption into the abdominal wall during menstrual flow is a common occurrence in 90% of menstrual females with patent fallopian tubes, even though it is only seen in those with hormonal or immunological issues [16,17]. The peritoneal fluid of women with endometriosis has higher amounts of macrophages, T-lymphocytes, and B-lymphocytes, which are more susceptible to apoptosis [18,19].

## Review

Clinical diagnosis of endometriosis

Endometriosis lesions are most frequently encountered in the following regions like fallopian tubes, uterus outer surface, ovaries, and the ligaments which surround the uterus. Lesions from endometriosis can range in size and frequently take the form of nodules or cysts. The majority of them are blue, black, and brown in color. They can, however, sometimes occasionally be white, red, or transparent [20,21]. Infertile women with minor or mild endometriosis have had the condition detected more frequently during in vitro fertilization (IVF) and embryo transfer (ET) cycles than those with moderate or severe endometriosis [1,22-24]. With a diagnostic age of 28 years, endometriosis predominantly affects women who are of reproductive age, which may be understood by the estrogenic milieu, which is strongly implicated in its genesis [25]. Extrauterine endometrial cells on laparoscopy are generally enough for histologic confirmation [26]. Cystic endometriomas might be reliably recognized utilizing transvaginal ultrasonography, while more modest endometrial implants are not detected [27,28].

Infertility and severe pelvic discomfort are the two major clinical risk factors for endometriosis [29-32]. The etiology of infertility in women with minor or mild endometriosis is less understood. However, it might be linked to a greater prevalence of malformed oocytes, faulty embryos, or unsuccessful implantation [33]. According to several studies, tall women with low Body Mass Index (BMI) appear at a greater risk of developing endometriosis because they have short menstrual cycles, are more likely to have cervical canalization problems, and decreased germ cell endowment [34]. Endometriosis has also been found to be less common among women who have been pregnant [29]. This might be owing to pregnancy's protective impact, or it could be due to endometriosis patients' lower fertility, as endometriosis risk is inversely associated with the number of term pregnancies [29,35,36].

Endometriosis was found in 19% of women with persistent pelvic discomfort at the point of surgery [37,38]. Dysmenorrhea, intermenstrual discomfort, and dyspareunia are common symptoms of pelvic pain. The most prevalent symptom is dysmenorrhea, which, while not predictive, is highly indicative of endometriosis in its severe form [39]. Dysmenorrhea is frequently progressive, with discomfort beginning before menstrual flow and lasting for the whole of the menstrual cycle and possibly for many days after that [40,41]. Endometriosis of the rectovaginal septum and cul-de-sac are common causes of dyspareunia [42]. Dysmenorrhea and dyspareunia are more likely to be caused by endometriosis if they appear after years of pain-free menses and coitus [43,44]. The overproduction of uterine prostaglandins aids pathogenesis. Urinary tract endometriosis can induce hematuria, urgency, dysuria, and frequency [45,46].

Dysmenorrhea

During menstruation, dysmenorrhea is a medical disorder characterized by discomfort around the pubic bone and lower abdomen [47,48]. Dysmenorrhea is related to a limitation of activity and leaves from school or work, precisely when it is severe [49]. Mental preparedness and suitable lifestyle changes, such as frequent physical activity, can help control dysmenorrhea [50]. Dysmenorrhea can be primary or secondary. Primary dysmenorrhea is a kind of dysmenorrhea that occurs in teenagers shortly after menarche and is caused by a lack of underlying macroscopic pelvic disease [51]. Early menarche, lengthier menstrual cycles, high menstrual flow, and a family history of dysmenorrhea are all risk factors [52,53].

Dyspareunia

Dyspareunia, or pain during penetration, occurs during sexual activity. Deep and superficial dyspareunia are the two most common kinds of dyspareunia [54]. Deep dyspareunia refers to the expansion of discomfort into the deeper regions of the vagina or lower pelvis, whereas superficial dyspareunia is restricted to the vulva or vaginal entrance. Profound penetration is typically correlated with deep dyspareunia [55]. Deep dyspareunia in endometriosis has a complicated etiology that might involve direct endometriotic lesions, concomitant diagnosis, and other factors [56]. Deep dyspareunia affects 50% of all female patients, a disorder that affects 10% of reproductive-age females and is linked to the poor sexual quality of life. Other forms of pelvic pain, psychiatric comorbidities, and concomitant pain diagnoses can occur in women with endometriosis [55,57].

Pathogenesis of endometriosis

Endometriosis pathogenesis suggests that the disease's etiology is complicated and multifaceted, involving genetic, hormonal, immunological, and environmental factors [58]. The pathogenesis of superficial endometriosis and the genetic factors that prevent ectopic lesions from being removed and allow for peritoneal remodeling may both be triggered by retrograde menstruation. These are critical for endometriosis lesion growth [59]. Endometriosis is spread by a change in the peritoneal fluid composition due to biological, hormone, and environmental factors [60,61]. Deep nodule tumors that are not generally eliminated during menstruation, might be quickly produced in a baboon model by transplanting basal endometrial tissue [62,63]. Endometriosis progresses due to genetic changes that damage the cell, since females with endometriosis have an abnormal endometrial cellular response, favoring extrauterine adhesion and proliferation [64]. Endometriosis has a genetic inheritance pattern that is believed to include numerous loci, as well as some chromosomal areas that have been linked to the endometriosis phenotype [65]. Genetic factors, both inherited and acquired, might predispose females to the adherence of abnormal endometrial tissues to the peritoneal epithelium as well as the resistance of immune clearing of these lesions [66]. Endometrial fragments that are still viable are pushed into the fallopian tubes by a pressure gradient caused by dyssynergic uterine contractions [67,68]. They can implant, develop, and infiltrate pelvic bones when they enter the peritoneal cavity [69,70]. Hyperalgesia is a symptom of nerve pain, which is generally caused by nerve damage or inflammatory stimulation. Endometriotic stroma cells typically invade sensory nerve fibers in deep endometriosis [71,72].

**Table 1:** Stages of endometriosis

Stage	Description
I	Isolated implants characterize this minimal disease. There is no adhesions present.
II	The peritoneum and ovaries are covered in superficial implants, which are a mild form of the disease. There were no significant adhesions seen.
III	Multiple implants, both superficial and highly intrusive, make up the modern disease. Adhesions on the ovaries and fallopian tubes are possible.
IV	Severe sickness is defined by several deep and superficial implants, massive ovarian endometriomas, and other symptoms. Typically, dense adhesions are present.

Methods

We focused on pelvic endometriosis in this study since it is the most prevalent kind of endometriosis, and it usually affects women throughout their reproductive years. PubMed and Google Scholar were used to conduct a review of the clinical diagnosis of endometriosis. In the titles or abstracts of papers, the search strategy contained the terms “endometriosis” and “clinical diagnosis.” The data from each clinical diagnosis document were extracted and categorized.

Result

In this study, there were 29 cases of endometriosis in total. The age range of the patients was 20 to 45 years, with a mean age of 29.4±7.7 and a median age of 28.8 years. The scar endometriosis (16 cases) involving the anterior abdominal wall at the lower segment cesarean section (LSCS) scar site, ovarian endometriosis (10 cases), urinary bladder endometriosis (two cases), and bowel endometriosis (one case) involving the sigmoid colon. According to the data, ovarian endometriosis and scar endometriosis are the two types of endometriosis that are most common in women aged 20 to 35. Scar endometriosis has been the most frequent kind of endometriosis in this research, accounting for 50% of all cases. In 14 of the 16 patients, there was a mass in the LSCS scar, which was identified by preoperative Fine Needle Aspiration Cytology (FNAC) in nine instances (56.25%) and histological analysis of excised tissues in seven cases (43.75%). The second most prevalent condition was ovarian endometriosis, which accounted for 37.5% of cases, with one instance being bilateral and another showing torsion. Bowel endometriosis with only one case involving the sigmoid colon accounted for 4.3% of cases, whereas two cases with urinary bladder endometriosis accounted for 8.3%.

Treatment of endometriosis

The goal of medical therapy for endometriosis has been to change the menstrual period hormonally to induce a pseudopregnancy, pseudo-menopause, or persistent anovulatory state [73]. The recommended daily dose of danazol for the treatment of endometriosis is 600-800 mg; even so, the dose has significant steroid side effects, including increased hair growth, changes in mood, an irreversible deepening of the voice, bad impacts on serum lipids, and, in rare cases, irreversible and life-threatening liver injury [74]. Endometriosis is typically treated with progesterone-based medications. They induce endometrial cells to decidualize, resulting in atrophy. Abnormal uterine bleeding, vomiting, breast discomfort, and depression are all possible side effects [75,76]. Hypoestrogenism's adverse effects include vaginal heavy bleeding, vaginal dryness, reduced libido, breast discomfort, sleeplessness, and depression [77]. Endometriosis has been treated with a variety of hormones and medicines. Hormone therapy often prevents ovulation by preventing the ovaries from releasing hormones, including estrogen. This could aid in reducing the rate of local development and activity of the endometrium and endometrial lesions. Because of their negative side effects, several hormones, such as methyltestosterone and estrogen, have been phased out. Other medications have been inadequately investigated, including clomiphene, tamoxifen, and the anti-progestational drug mifepristone [78]. Laparoscopic surgery is used to surgically diagnose endometriosis. A laparoscope, a narrow viewing tube, is inserted into the belly through a tiny incision during laparoscopy. For a second entrance for the surgical tools, a second incision may be created in the lower abdomen. Your doctor can view the outside of the ovaries, uterus, fallopian tubes, and other organs up close using a laparoscope. Surgical tools for removing scar tissue or obtaining tissue samples can also be attached to the laparoscope [79]. For ovarian cysts greater than 4 to 5 cm in diameter, laparoscopic endometrioma removal is suggested [80]. Endometriosis has been treated with a range of devices, from monopolar cautery and scissors to a wide variety of lasers and ultrasonic scalpels [81]. Endometriosis pain is treated surgically by interrupting the brain circuits that carry pain signals [82]. Various considerations should be taken, including the best method of gaining access to the pelvis and abdomen, which can be accomplished by laparoscopy or laparotomy. Laparoscopy is less expensive and takes less time to recuperate. Magnetic resonance imaging (MRI) scan not only shows morphologic defects in the bladder but can also identify other common areas, such as the uterosacral joints, where ultrasonography is less accurate [83].

Discussion

Endometriosis affects people and cultures all over the world; however, there are significant delays in diagnosis. More illness awareness in inpatient healthcare should result in earlier diagnosis, less suffering, and increased productivity [84,85]. Gravidity, endometriosis in the family, dyspareunia, tiredness, dysmenorrhea, pelvic surgery, diarrhea, pelvic discomfort, and premenstrual spotting are all risk factors for endometriosis [86]. Several explanations for the connection between endometriosis and infertility have been claimed, but despite substantial investigation, no conclusion has been achieved. These mechanisms include distorted pelvic anatomy, altered peritoneal function, endocrine, and ovulatory abnormalities, and altered hormonal and cell-mediated functions in the endometrium [87,88]. Increased obstetric surgery results in the direct mechanical implantation of endometrial cells, which is one of the major risk factors for scar endometriosis [89].

When a patient experiences dysmenorrhea after experiencing pain-free menstrual cycles for years, endometriosis should be suspected [90]. Endometriomas having a maximal diameter of 3-4 cm are best treated through transvaginal cystectomy. Less than 1% of women develop ovarian endometriosis malignancy, which most frequently manifests as endometrioid/clear cell carcinoma [91]. Between 1% and 5% of women with endometriosis have urinary tract involvement, with the urinary bladder being the most commonly affected, followed by the ureter, urethra, and kidney [92,93]. In 3%-37% of instances, endometriosis was known to have an impact on the digestive system, with the recto-sigmoid colon as the most often affected area [94]. Although comparable findings have been documented in cancer patients, the link between diagnostic delay and health care funding for endometriosis is unique [95-97]. The majority of endometriosis patients have normal pelvic findings; hence a laparoscopy is required to make a conclusive diagnosis. It is likely that ultimately a combination of biochemical indicators and clinical evaluation will lessen the requirement for surgical confirmation, even if no single laboratory test has demonstrated proven clinical usefulness [98,99]. Endometriosis is more frequent among Caucasian, middle-aged, upper-class women who are ambitious. This could happen because these women have more access to medical treatment and diagnostic procedures like laparoscopy [100]. This might be because these women have easier access to medical treatment and diagnostic testing like laparoscopy [101,102]. As endometriosis frequently exhibits symptoms that resemble those of other disorders and cause a diagnostic delay, early suspicion of the condition is crucial for prompt diagnosis. In addition to a patient's medical history, secondary centers with access to further investigations may need to be referred from the main health care level [103,104].

Ovarian Endometriosis

Ovarian endometriomas account for 35% of all benign ovarian cysts and are seen in 17%-44% of female patients [105]. Endometrial cell growth may be induced by ovarian follicular fluid [106]. Left ovary endometriomas are more prevalent than right ovary endometriomas [107]. The amount of normal ovarian in the swollen ovarian cortex was decreased in endometrioma patients' ovarian cortical tissue, a result not seen in other benign cysts to a similar extent [108]. The ovary with endometrial cysts exhibits lower reactivity to exogenous gonadotropin activation, with more follicular atresia, and a higher rate of follicular atrophy [109]. The most prevalent histologic forms of ovarian cancer resulting from ovarian endometriosis are endometrioid adenocarcinoma and clear cell carcinoma [110,111].

Scar Endometriosis

Scar endometriosis occurs when endometrial tissue is implanted directly in scars during surgery [112]. Other surgical disorders such as hematoma, hernia, scar tissue, neuroma, abscess, granuloma, or even metastatic cancer can easily be coupled with scar endometriosis [113,114]. The presence of morphological and physiological stroma and endometrial gland beyond the uterine cavity is a persistent gynecologic condition [115]. The kidneys, pleura, bladder, lungs, colon, lymph nodes, omentum, and abdominal wall are all common locations for extra pelvic endometriosis [116].

Urinary Bladder Endometriosis

Extragenital endometriosis most commonly affects the gut and urinary system [117]. The urinary bladder is a rare location of endometrial localization, with just around 1% of people that are suffering from the illness having lesions affecting the urine system [118]. To avoid kidney function loss, urinary tract endometriosis must be diagnosed and treated early. For women who complain of bladder pain but have a negative urine test, the doctor may not examine if the discomfort is cyclic or whether bladder endometriosis is a possibility. Endometriosis in the urinary system is uncommon, occurring in about 1%-6% of females with initially diagnosed endometriosis [119].

## Conclusions

Long-term conditions like endometriosis with symptoms like dyspareunia, dysmenorrhea, dysuria, dyschezia, and chronic pelvic pain (CPP) vary based on the organ affected, but not the illness itself. For early identification and management of bladder, ovarian, and intestine endometriosis, a higher index of concern is essential to improve the quality of life and decrease infertility. Dysmenorrhea is a fairly prevalent condition in adolescent girls, as they suffer from a variety of mental and physical symptoms that negatively impact their quality of life. Scar endometriosis has been the most common site of endometriosis in this research, with 50% of cases developing solely at the LSCS scar site. Minimal peritoneal endometriosis and also the most severe phases of the disease can be treated with laparoscopic surgery. Therefore, women with endometriosis are at a higher risk of miscarriage, and their embryo quality suffers.
